# SDF-1α-induced dual pairs of E-selectin/ligand mediate endothelial progenitor cell homing to critical ischemia

**DOI:** 10.1038/srep34416

**Published:** 2016-10-07

**Authors:** Zhao-Jun Liu, Runxia Tian, Yan Li, Leiming Zhang, Hongwei Shao, Cuixia Yang, Omaida C. Velazquez

**Affiliations:** 1 Department of Surgery, University of Miami School of Medicine, Miami, 33136, USA; 2 Yantai University, School of Pharmacy, Shandong 264005, China; 3 Department of Molecular Biology Laboratory, Shanghai Sixth People’s Hospital, Shanghai Jiaotong University, Shanghai, 200233, China

## Abstract

Homing of endothelial progenitor cells (EPC) to the ischemic tissues is a key event in neovascularization and tissue regeneration. In response to ischemic insult, injured tissues secrete several chemo-cytokines, including stromal cell-derived factor-1α (SDF-1α), which triggers mobilization and homing of bone marrow-derived EPC (BMD-EPC). We previously reported that SDF-1α-induced EPC homing is mediated by a panel of adhesion molecules highly or selectively expressed on the activated endothelium in ischemic tissues, including E-selectin. Elevated E-selectin on wound vasculature serve as docking sites for circulating EPC, which express counterpart E-selectin ligands. Here, we show that SDF-1α presented in wound tissue and released into circulation can act both locally and remotely to induce ischemic tissue endothelium and BMD-EPC to express both E-selectin and its ligands. By performing BM transplantation using E-selectin^−/−^ and E-selectin^+/+^ mice as the donors and recipients respectively, we demonstrate that upregulated dual E-selectin/ligand pairs reciprocally expressed on ischemic tissue endothelium and BMD-EPC act as double-locks to secure targeted EPC- endothelium interactions by which to facilitate EPC homing and promote neovascularization and tissue repair. These findings describe a novel mechanism for BMD-EPC homing and indicate that dual E-selectin/ligand pairs may be effective targets/tools for therapeutic neovascularization and targeted cell delivery.

Targeted homing of endogenously mobilized stem/progenitor cells and exogenously infused therapeutic stem cells from the circulation to injured tissue is a crucial event in stem cell-based therapy and stem cell-mediated tissue repair/regeneration^1^,^2^,^3^. Ischemic tissue-specific homing is triggered by a dynamic and coordinated multi-step paradigm involved in chemo-cytokines induced by low-oxygen sensor hypoxia-inducible factor-1α (HIF-1α). Among these chemo-cytokines, SDF-1α is a potent chemoattractant for trafficking and migration of stem and progenitor cells. SDF-1α can exert its role through binding with its cognate receptor C-X-C chemokine receptor 4 (CXCR4), which is expressed on the surface of EPC^4^,^5^ and it may function as a sensor for circulating EPC cruising through sites on the microvasculature in ischemic tissue that have an SDF-1α gradient. Inhibition of the SDF-1α/CXCR4 interaction is capable of blocking, although partially, the homing of progenitor/stem cells to the ischemic myocardium^6^,^7^. Because SDF-1α is a secreted soluble factor, even if it binds to CXCR4 on the surface of the circulating EPC, it would not directly mediate attachment of EPC to the endothelium. Under the shear force of the blood flow, circulating EPC would unlikely to be anchored on the endothelium in ischemic tissue. Although an early study reported that SDF-1α could form complexes *in vitro* with heparin sulfate, a glycosaminoglycan (GAG) found ubiquitously on the cell surfaces and in the extracellular matrix^8^,^9^, it is unknown whether SDF-1α can interact with cell-membrane-bound-GAG *in vivo*, especially with GAG on the endothelium under shear force. We previously reported a novel mechanism whereby SDF-1α regulates EPC homing. We demonstrated that SDF-1α can specifically induce the expression of E-selectin in luminal endothelial cells (EC), and elevated E-selectin is responsible for mediating EPC homing by enhancing adhesion of circulating EPC to luminal EC in ischemic tissue and subsequent transendothelial migration and extravasation. These effects resulted in a significant enhancement of wound neovascularization and repair in a mouse hind limb ischemia model^10^.

E-selectin is an inducible vascular adhesion molecule prevalently expressed on activated EC in response to inflammation and injury. Its role in the recruitment of circulating leukocytes^11^,^12^, hematopoietic stem cells (HSC)^13^, and EPC^10^,^14^,^15^ takes place mainly through association with its counterpart ligands, including PSGL-1/CD162, ESL-1 and CD44, expressed on these circulating cells. Recent studies, however, have begun to indicate the presence of E-selectin on murine BMD-EPC, even though the role of E-selectin on EPC has not yet been established^14^. The expression of PSGL-1/CD162 on the endothelium^16^, in particular on the endothelial lining of atherosclerotic coronary arteries has been reported^17^. This suggests a potential role in the formation of the inflammatory infiltrate in arterial wall lesions. In this way, reverses in the pattern of expression of E-selectin on EPC and E-selectin ligand on endothelium may also be involved in mediating EPC-EC interactions and EPC homing. Indeed, we have recently demonstrated that levels of CD44 are upregulated on the endothelium of acute wound bed capillaries, and the elevated CD44 function as docking sites for wound tissue homing of infused exogenous cells which are installed with soluble E-selectin on cell surface^18^.

This study builds upon previous findings and shows that SDF-1α administrated and elevated in ischemic tissue can induce expression of E-selectin and its ligands in both local ischemic wound endothelium and remote BM EPC and circulating EPC. Upregulated dual E-selectin/ligand pairs reciprocally expressed on activated endothelium in ischemic wound tissue and mobilized BMD-EPC mediate EPC-EC interactions to promote targeted EPC homing, increased wound neovascularization, and faster tissue repair.

## Results

### SDF-1α supplied to ischemic wound tissue has a remote role in inducing E-selectin expression on BMD-EPC

**Figure 1:**
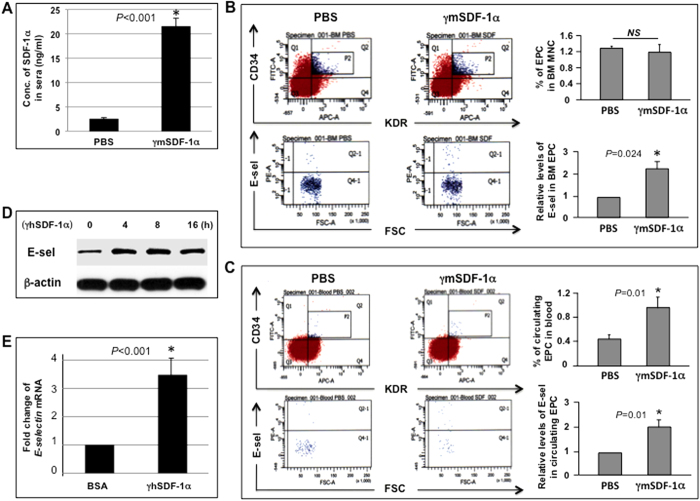
SDF-1α induces E-selectin expression in EPC. (**A**) Levels of mSDF-1α in sera 4 h after wound bed injection of γmSDF-1α vs. PBS (n = 8 mice/group). (**B**) Measurement of levels of E-selectin in BM EPC by flow cytometry. BMC were incubated with Abs against CD34, KDR, and E-selectin or isotype-matched control Abs. EPC (CD34^+^/KDR^+^) were gated (P2), counted, and analyzed for levels of E-selectin (Q2-1). Bar graphs show % of EPC in BM mononuclear cells and relative levels of E-selectin in BM-EPC. Levels of E-selectin in EPC from mice injected with PBS were established as “1” and relative levels of E-selectin in EPC from mice injected with γmSDF-1α were normalized accordingly (n = 8 mice/group). (**C**) Measurement of % of EPC in peripheral blood MNC and relative levels of E-selectin in circulating EPC as described in (**B**) (n = 8 mice/group). (**D**) Immunoblotting analysis of E-selectin expression upon γhSDF-1α (100 ng/ml) stimulation in human EPC at various time points. β-actin served as a loading control. (**E**) Human EPC were stimulated with γhSDF-1α or BSA for 4 h, and total RNA was extracted. Expression of extracellular matrix and adhesion molecules were analyzed using *RT2-PCRArray*. Expression of *E-selectin* was upregulated upon γmSDF-1α stimulation. Levels of *mRNA* in BSA-treated EPC were established as “1” and compared to those in γmSDF-1α-treated EPC. Experiments were repeated three times in (**D**) and (**E**). Data are analyzed by 2-tailed Student’s *t*-test and presented as mean ± SEM.

Wound tissue SDF-1α, either secreted by local tissue cells in response to ischemic insult or therapeutically administrated, can be released into circulation. To study the remote effect of local SDF-1α in ischemic diabetic wound tissue on EPC resided in their BM niche and in circulation, ischemic hindlimb wounds were made in *NOD* mouse. Because the tissue levels of SDF-1α in diabetic murine wounds are significantly lower than in healthy tissue, as indicated in a previous study^19^, exogenous recombinant mouse SDF-1α (γmSDF-1α) (25 μg/kg, this dosage was determined in our previous study^10^) was injected into the wound bed. Equal volumes of solvent (PBS) were injected into wound bed in the control group (n = 8/group). Concentrations of SDF-1α in sera collected from the tail vein 4 h later were measured by ELISA. Local wound injection of γmSDF-1α resulted in elevated serum levels of SDF-1α compared with PBS injection in control mice (Fig. 1A), indicating the release of locally administrated γmSDF-1α into circulation. Elevated SDF-1α in sera can either stimulate circulating EPC or reach BM to stimulate EPC present in BM. Both BM and peripheral blood were harvested 24 h after wound tissue SDF-1α injection. Cells were incubated with FITC-CD34, APC-KDR, and PE-E-selectin antibodies (Abs). E-selectin levels in the gated EPC (EPC are defined as CD34^+^/KDR^+^ herein) fraction were measured using flow cytometry analysis. In response to elevated systemic levels of SDF-1α, expression of E-selectin in EPC from BM (Fig. 1B) and peripheral blood (Fig. 1C) was significantly increased, showing that local supplementation of SDF-1α in ischemic wound tissue can have a remote effect on expression of E-selectin in BM and circulating EPC. SDF-1α also appeared to promote EPC mobilization in that there were approximately 1-fold more EPC in peripheral blood (Fig. 1C) in response to locally administrated SDF-1α. The comparable amounts of EPC in BM (Fig. 1B) suggested a quick self-renewal of EPC to maintain a stable BM EPC pool after EPC mobilization.

To confirm these observations, we conducted immunoblotting analyses and confirmed upregulation of E-selectin in recombinant human SDF-1α (γhSDF-1α)-stimulated human EPC *in vitro* (Fig. 1D). A RT^2^ Profiler™ PCR array was made to examine gene expression profile of expanded panel of cell adhesion molecules and extracellular matrix in EPC in response to SDF-1α. Of the 84 genes, 3 were downregulated (−) and 22 were upregulated (+) (cut-off value: < or >2-fold) (Supplemental Table 1), and 59 were unaltered. Notably, expression of the *E-selectin* gene was increased about 3.48-fold upon SDF-1α stimulation (Fig. 1E). PCR array data offered additional candidates for future elucidation of the mechanisms underlying SDF-1α-triggered BMD-EPC homing and mobilization.

Collectively, these data demonstrated that local administration of SDF-1α to ischemic wounds could have a remote effect on expression of E-selectin in BM and circulating EPC, and mobilization of BMD-EPC.

### SDF-1α induces cultured EC and mouse wound endothelium to express E-selectin ligands *in vitro* and *in vivo*

**Figure 2:**
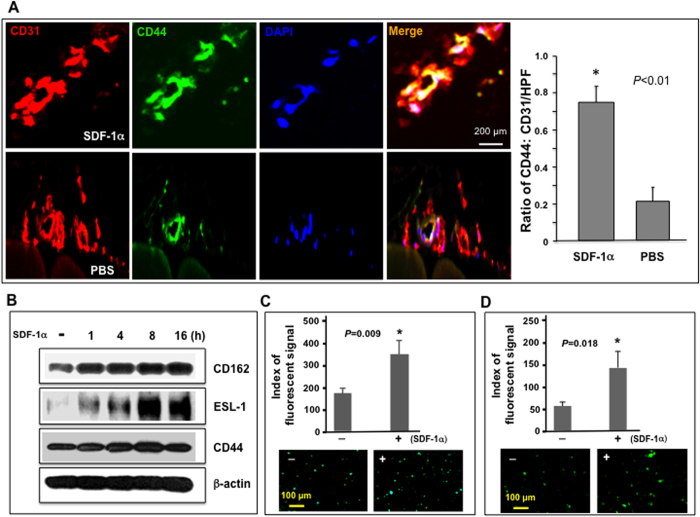
SDF-1α induces expression of active form of E-selectin ligands in EC. (**A**) Increased expression of E-selectin ligand, CD44, in luminal EC of ischemic hindlimb wounds injected with γmSDF-1α compared to PBS injected non-wounded hindlimb in *NOD* mice (n = 8 mice/group). (**A**) *right*: Co-expression (yellow) of CD44 (green) and CD31 (red) in vessels was detected by immunostaining. Representative images are shown (images of isotype-matched non-specific control Ab are not shown). *Left*: Quantification of CD44 expression in vessels. Data are presented as mean ± SD of ratio of CD44:CD31 signals from 5 random selected sections of high power field (LPF, X 20) of each wound sample. CD31 signal was established as “1” in each section and relative amount of CD44 signal was normalized accordingly. (**B**) Immunoblotting analysis of SDF-1α-induced expression of three E-selectin ligands in HMVEC at various time points (γhSDF-1α: 100 ng/ml). β-actin is used as loading control. Experiments were repeated three times and similar results were obtained. (**C**) Binding of FITC-conjugated HECA452 to HMVEC stimulated with γhSDF-1α or BSA. Fluorescent signals were quantified (*top*). Representative fluorescent images were exhibited (*bottom*). (**D**) Binding of FITC-conjugated HECA452 to human EPC stimulated with γhSDF-1α or BSA (100 ng/ml). Fluorescent signals were quantified (*top*). Representative fluorescent images were exhibited (*bottom*). Data are analyzed by 2-tailed Student’s *t*-test and presented as mean ± SEM of fluorescent signals based on triplicate wells in each condition and totally three independent experiments in (**C**,**D**).

To determine whether wound endothelium expresses counterpart ligands of E-selectin in response to locally administrated γmSDF-1α in wound tissue, the expression of the E-selectin ligand CD44 was specifically examined in the microvasculature of ischemic hindlimb wounds of *NOD* mouse 24 h after wound bed injection of γmSDF-1α, because the presence of PSGL-1/CD162 on injured or inflamed endothelium has already been established^17^. We observed that CD44 expression in luminal EC was markedly higher in ischemic diabetic wounds than endothelium in the control hindlimb tissue (non-ischemia, PBS injection) of the same mouse (n = 8/group) (Fig. 2A), demonstrating that elevated SDF-1α in wound tissue caused the local endothelium to increase expression of the E-selectin ligand CD44.

Immunoblotting assays were performed to validate upregulation of E-selectin ligands by SDF-1α in EC. Increased protein expression of three E-selectin ligands, CD44, CD162, and ESL-1, in human microvascular endothelial cells (HMVEC) upon SDF-1α stimulation was confirmed (Fig. 2B).

E-selectin ligands can be expressed as unmodified inactive form or modified active form. The active form can associate with E-selectin. To determine whether SDF-1α-induced E-selectin ligands are in the inactive or active form, a cell surface E-selectin ligand binding assay was conducted to determine whether FITC-conjugated HECA452, which is mAb directed against a sialyl Lewis X (sLeX) epitope on active E-selectin ligand CD162^20^ and CD44^21^, can associate with SDF-1α-induced E-selectin ligands on HMVEC and EPC. It had already been demonstrated that SDF-1α also induces E-selectin ligands expressed in EPC^10^. Approximately 1-fold and 1.4-fold more FITC-HECA452 was found to be bound to SDF-1α-stimulated HMVEC (Fig. 2C) and EPC (Fig. 2D) compared to non-stimulated HMVEC and EPC, respectively, indicating that SDF-1α-induced E-selectin ligands expressed on HMVEC and EPC are modified as active form, which can be associated with HECA452. These experiments demonstrated that SDF-1α could cause HMVEC and EPC to express three E-selectin ligands, CD44, CD162, and ESL-1, which are likely to be modified into an active form.

Therefore, SDF-1α-induced E-selectin ligands expressed on the ischemic diabetic wound endothelium may serve as docking adhesion molecules to mediate anchorage of E-selectin^+^ circulating cells, for example, SDF-1α-triggered circulating EPC, on the activated endothelium.

### Up-regulated E-selectin on EPC and E-selectin ligands on EC are responsible for mediating SDF-1α-induced EPC-EC interactions

**Figure 3:**
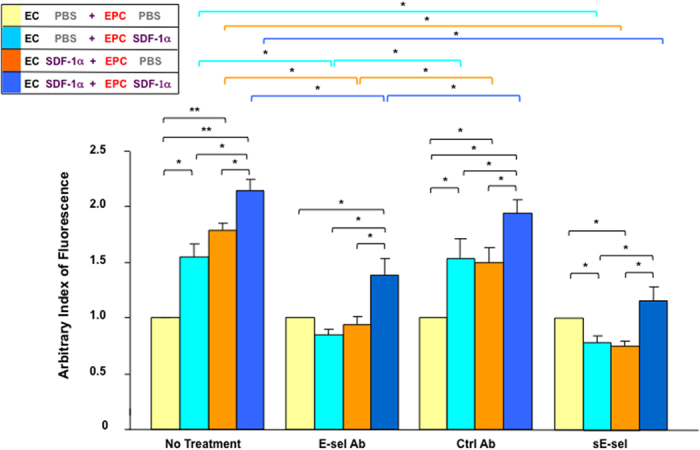
SDF-1α-induced E-selectin/ligands are responsible for mediating EC-EPC adhesion *in vitro*. SDF-1α increases adhesion of Dil-Ac-LDL^+^ human EPC to the HMVEC monolayer. SDF-1α-induced cell-cell adhesion between EPC and HMVEC depends upon E-selectin expressed on EPC and E-selectin ligands expressed on HMVEC, as indicated by the fact that treatment of SDF-1α-stimulated HMVEC monolayers with sE-sel or treatment of SDF-1α-stimulated Dil-Ac-LDL^+^ EPC with E-selectin-neutralizing Ab can significantly inhibit EPC-HMVEC interaction. Four combinations of EPC-HMVEC interaction are illustrated in the bottom. Data are analyzed by two-way ANOVA test and presented as mean ± SEM of fluorescent signals (Dil) based on triplicate wells in each condition and totally three independent experiments. *P < 0.05; **P < 0.01.

We previously reported that SDF-1α induced EPC-EC adhesion *in vitro*, which is mediated by up-regulated E-selectin on EC and E-selectin ligands on EPC^10^. To determine whether SDF-1α-induced E-selectin on EPC and E-selectin ligands on EC, which have been identified in the current study, are also involved in mediating SDF-1α-enhanced EPC-EC adhesion, the effect of antagonists against E-selectin and its ligands on inhibition of SDF-1α-enhanced EPC adhesion to EC monolayer was tested *in vitro*. Human EPC were pre-labeled with Dil-Ac-LDL. Dil-Ac-LDL^+^ EPC were detached from dishes and cultured in agarose gel-coated dishes to prevent adhesion. These EPC were then stimulated with γhSDF-1α or BSA. After 4 h, Dil-Ac-LDL^+^ EPC were replaced with EGM2 medium containing either E-selectin neutralizing Ab or isotype-matched control Ab (2 μg/ml) and incubated for 15 min at 37 °C. HMVEC were grown as a monolayer and stimulated with γhSDF-1α or BSA for 4 h. The stimulated HMVEC monolayer was re-cultured in EGM2 media containing either soluble E-selectin (sE-sel) or BSA (2 μg/ml) and incubated for 15 min at 37 °C. After washing both EPC and HMVEC with PBS to remove un-associated E-selectin-neutralizing Ab or sE-sel, non-adherent Dil-Ac-LDL^+^ EPC were collected from agarose-gel-coated dishes and added to the wells containing the HMVEC monolayer to allow EPC-EC interactions. After 30 min, unbound EPC were washed out by PBS (twice) and Dil-Ac-LDL^+^ EPC associated with HMVEC-monolayers were measured using a fluorescence scanner. As in previous reports, SDF-1α-stimulated EPC and HMVEC monolayers showed significantly more EPC-EC binding capability than un-stimulated control cells (Fig. 3). Combined treatments of EC and EPC with SDF-1α increased EPC-EC interactions. There were more EPC associated with EC monolayers compared to single treatment (EC or EPC treated with SDF-1α). It suggested that stimulation of both EC and EPC with SDF-1α induce both types of cells to express elevated levels of E-selectin/lignad pairs that mediate stronger EPC-EC interactions. Both treatment of SDF-1α-stimulated EPC with E-selectin-neutralizing Ab and treatment of SDF-1α-stimulated HMVEC monolayers with sE-sel alone significantly inhibited EPC-EC interactions, but control Ab and BSA (Supplemental Figure 1) had no significant effect. Combination of E-selectin-neutralizing Ab and sE-sel showed even stronger inhibitory effect on EPC-EC interaction (Supplemental Figure 1). Overall, these results demonstrated that upregulated E-selectin on EPC and E-selecin ligands on EC are also responsible for mediating SDF-1α-enhanced EPC-EC interactions.

### E-selectin on both EPC and wound endothelium are required for mediation of SDF-1α-induced EPC homing

**Figure 4:**
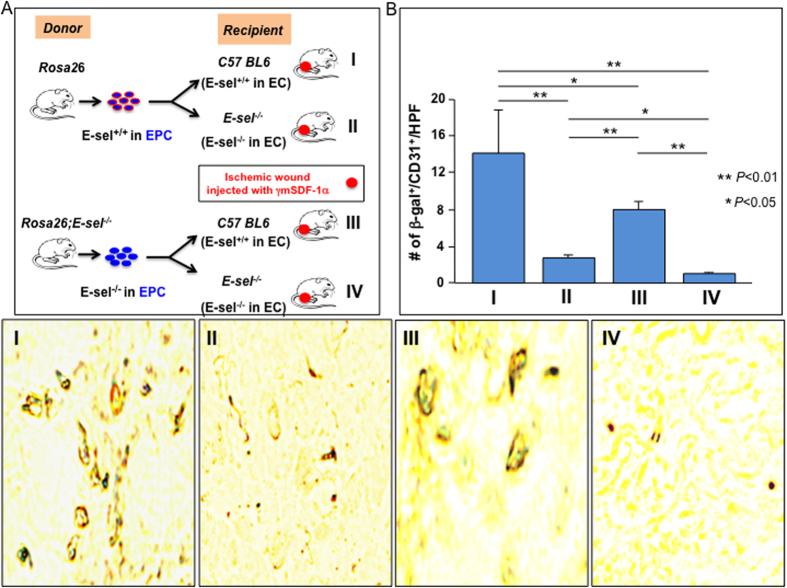
E-selectin/ligands are essential for EPC homing to ischemic wound tissue. (**A**) Illustration of BMT in various conditions (I–IV). (**B**) Decreased homing of E-sel^−/−^-EPC into ischemic wounds where endothelium do not express E-selectin. *Top*: Quantitative data of β-gal^+^/CD31^+^ cells/HPF. Positive cells were counted from 5 randomly selected fields in each section. Five sections of each wound sample were examined. Data are analyzed by two-way ANOVA test and presented as mean ± SEM (n = 6 mice/group). *Bottom*: Representative images of *X-gal* (blue) and anti-CD31 staining (brown) of frozen samples of wound tissues.

To assess the involvement of SDF-1α-induced E-selectin on both EPC and wound endothelium in mediating EPC homing to wound tissue, syngeneic BM transplantation (BMT) was used to assess the homing capability of EPC derived from two types of donor mice, *Rosa26*^+/−^ vs. *Rosa26*^+/−^*;E-sel*^−/−^ in which LacZ^+^-BM cells, including EPC, either expressed or did not express E-selectin in response to SDF-1α (E-sel^+/+^-EPC vs. E-sel^−/−^-EPC), to wound tissues of two types of recipient mice, *C57BL6* vs. *E-sel*^−/−^ in which all tissue cells, including wound endothelium, expressed or did not express E-selectin (E-sel^+/+^-EC vs. E-sel^−/−^-EC). BM of γ-irradiated recipient mice (*C57BL6* vs. *E-sel*^−/−^) were cross-reconstituted using LacZ^+^-BM cells from donor mice (infusion of LacZ^+^-BM cells from *Rosa26*^+/−^ vs. *Rosa26*^+/−^*;E-sel*^−/−^ mice (n = 6/group) into recipient mice (*C57BL6* vs. *E-sel*^−/−^, n = 12/group)). Recipient mice were subjected to femoral ligation to create unilateral hindlimb ischemia and subsequent bilateral 4 mm cutaneous excision. γmSDF-1α was administered to wound bed. Recipient mice were killed after 7 days, and half of mice (n = 6/group) were used to harvest wound tissues to perform IHC and the remaining mice (n = 6/group) were used for Dil-perfusion to measure neovascularization (see below). LacZ^+^-EPC that were recruited to wound tissues and incorporated into blood vessels were detected using double staining with *X*-*gal* (blue) and anti-CD31 (brown). We observed significantly less LacZ^+^/CD31^+^-EPC in blood vessels of ischemic wounds in the recipient *C57BL6* mice which were transplanted with BMC from *Rosa26*^+/−^*;E-sel*^−/−^ mice than that from *Rosa26*^+/−^ mice (Fig. 4), indicating that E-selectin expressed on EPC is required for mediating SDF-1α-induced EPC homing. Consistent with previous observations, there were fewer LacZ^+^/CD31^+^-EPC in blood vessels of ischemic wounds in the recipient *E-sel*^−/−^ mice than in those of *C57BL6* mice (Fig. 4), confirming that E-selectin expressed in wound endothelium is also essential to mediation of SDF-1α-induced EPC homing.

### E-selectin on both EPC and EC is essential to mediation of SDF-1α-induced neovascularization and re-perfusion in ischemic limb

The role of SDF-1α-induced E-selectin on EPC and on wound endothelium in regulation of neovascularization in wound tissue and perfusion in ischemic limb was explored. Neovascularization was evaluated by whole-body blood vessel Dil perfusion and subsequent laser scanning confocal microscopy in wound tissues harvested from recipient mice (n = 6/group) on day 7. Laser Doppler perfusion imaging (LDI) was used to confirm post-operative limb ischemia and quantify the spontaneous restoration of hindlimb blood flow over time (n = 12/group).

**Figure 5:**
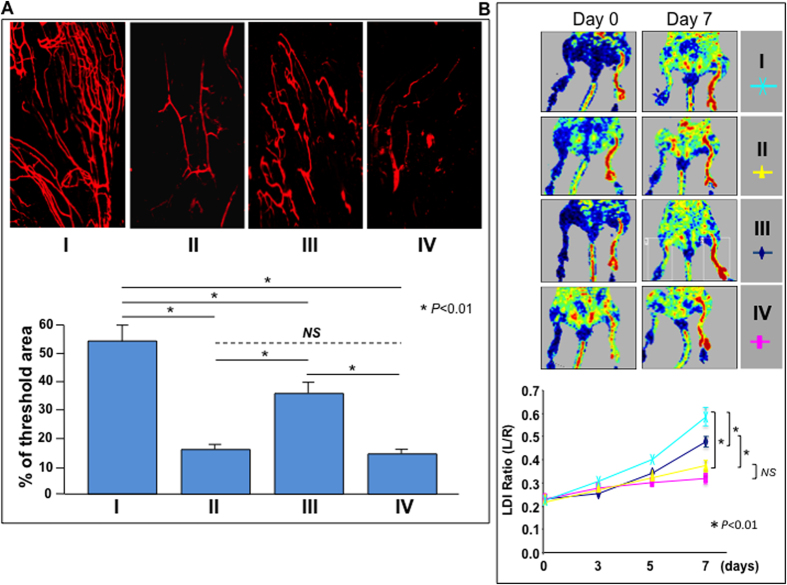
E-selectin/ligands are required for EPC-dependent blood vessel formation. (**A**) Wound blood vessel perfusion with Dil dye. *Upper*: representative images of Dil-stained wound blood vessels detected by confocal laser scanning photography at day 7 are shown for each type of recipient mice (I–IV as illustrated in Fig. 4A). *Lower*: Quantification of vessel density in the entire area of residual wounds on day 7, as percent fluorescence. (**B**) *Upper*: representative images of non-invasive LDI measurements showing spontaneous restoration of blood flow into ischemic hindlimbs after femoral artery ligation/excision in different recipient mice (I–IV as illustrated in Fig. 4A). *Lower*: Quantitative data of LDI measurements. Ratio of ischemic versus normal hindlimb between two groups of mice at various time points. For both (**A**,**B**), data are analyzed by two-way ANOVA test and presented as mean ± SEM from each group (n = 6/group).

There was significantly higher blood vessel density in ischemic wounds (Fig. 5A) and increased mean flux measurements in the ischemic limb (Fig. 5B) of recipient *C57BL6* mice transplanted with BMC (containing LacZ^+^-EPC) from *Rosa26*^+/−^ mice compared to that from *Rosa26*^+/−^*;E-sel*^−/−^ mice, indicating that E-selectin expressed on EPC is required for mediation of SDF-1α-induced neovascularization. Consistent with previous observations, there were fewer blood vessels within wounds (Fig. 5A) and decreased mean flux measurements in the ischemic limbs (Fig. 5B) in recipient *E-sel*^−/−^ mice than in *C57BL6* mice, confirming that E-selectin was expressed in wound endothelium and essential to mediation of SDF-1α-induced neovascularization and reperfusion of the ischemic limbs.

### SDF-1α-induced E-selectin in both EPC and EC are critical for wound healing

**Figure 6:**
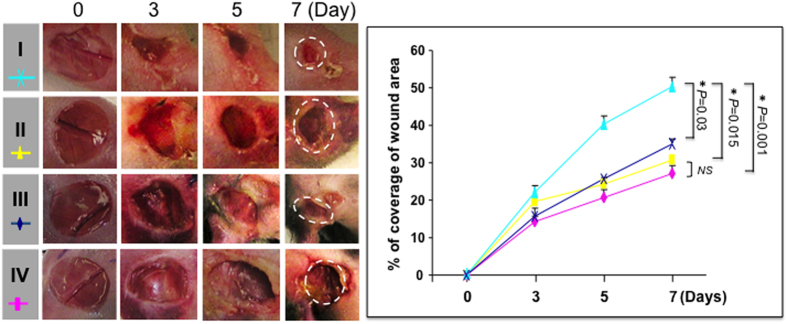
E-selectin/ligands are required for EPC-dependent wound healing. *Left*: representative images of wound healing in different recipient mice (I-IV as illustrated in Fig. 4A). Deletion of E-selectin delayed wound healing. *Right*: quantitative wound closure rates in in different recipient mice. Data are analyzed by two-way ANOVA test and presented as percentage wound closure (recovery), mean ± SEM from each group (n = 6/group).

The role of SDF-1α-induced E-selectin on EPC and on wound endothelium,in the regulation of wound healing was investigated. Wound areas were measured using daily digital photography until day 7 and calculated using ImageJ software. A delayed healing rate was observed in the recipient *C57BL6* mice, which were transplanted with BMC from *Rosa26*^+/−^*;E-sel*^−/−^ mice compared to that from *Rosa26*^+/−^ mice (Fig. 6), indicating that E-selectin expressed on EPC is required for mediating SDF-1α-induced wound healing. Similarly, healing rate in recipient *E-sel*^−/−^ mice was slower than in *C57BL6* mice (Fig. 6), confirming that E-selectin expressed in wound endothelium is also relevant to mediation of SDF-1α-induced wound healing.

## Discussion

Luminal EC form a natural barrier between the blood and surrounding tissue. Under steady-state physiological conditions, luminal EC are mostly quiescent and form a tight, impermeable barrier. Under pathological conditions, such as tissue ischemia, inflammation, and tumors, a variety of cytokines/chemokines, such as SDF-1α, TGF-ß, and IL-1, are produced and released into tissue, and the local tissue endothelium is stimulated by these soluble factors. It causes upregulation and activation of a unique panel of cell adhesion molecules (CAMs), including selectins and integrins, in the endothelium within the local tissue. This causes the local endothelium to switch from a tight impermeable to permeable and “sticky” status. These adhesion molecules act as docking sites and mediate tethering of circulating inflammatory cells and stem/ progenitor cells, including BMD-EPC. The anchored circulating cells undergo rolling, tight adhesion to the endothelium and subsequent transendothelial migration, extravasation from permeable capillaries and infiltration into diseased tissues. We previously demonstrated that SDF-1α upregulates E-selectin expression on the local wound endothelium and induces EPC to express E-selectin ligands^10^. The current study extend these findings and show that SDF-1α elevated or supplied in ischemic wound tissue has not only local but also remote effects, when it is released into circulation, on the expression of both E-selectin and its cognate ligands in wound endothelium and BMD-EPC. Upregulated dual E-selectin/ligand pairs reciprocally expressed in activated endothelium in wounds and mobilized BMD circulating EPC act as double locks, securing EPC-EC interactions by which to enhance selective homing of EPC to ischemic wound tissue, which is essential to neovascularization and tissue repair. In this way, the current findings provide profound and novel insight into the molecular mechanisms underlying the biological effects of SDF-1α on EPC homing and suggest that E-selectin and its ligands may be suitable targets and tools for therapeutic manipulation of targeted EPC homing. Full molecules, fragments, and binding epitopes of E-selectin and its ligands may also be utilized to direct therapeutic stem cells, for instance, mesenchymal stem cells (MSC), homing to sites of ischemic lesions for cell-based therapy.

It is worth mentioning that there still remain debate with respect to the definition and function of EPC in the field. Murine EPC are defined as CD34^+^/KDR^+^ (in flow cytomery analysis) and LacZ^+^/CD31^+^ (in *in vivo* bone marrow transplantation experiments) in our study although these cells may also be generally referred as pro-angiogenic bone marrow-derived mononuclear cells. BM-derived circulating progenitor/stem cells, including EPC, can be incorporated into newly formed capillaries, enhance neovascularization after hind limb ischemia and improve tissue function after ischemic injury. Alternatively, recruited progenitor/stem cells, including EPC, may also promote neovascularization and tissue regeneration by releasing soluble factors, which act in a paracrine manner to support local angiogenesis and mobilize tissue residing progenitor cells.

The current study identified SDF-1α as a trigger to induce dual E-selectin/ligand pairs (all three types of ligands) to be reciprocally expressed on both activated endothelium in wound tissue and BMD-EPC. SDF-1α-induced ligands were found to be functional on both EPC and endothelium because they undergo post-translational modification (glycosylation), as indicated by increased association with HECA452, which specifically recognizes sLeX epitope on active PSGL-1/CD162 and CD44^20^,^21^. In addition, SDF-1α can mobilize BMD-EPC into circulation, although the precise role of induced E-selectin/ligand pairs in EPC mobilization is unclear. In this way, SDF-1α produced or presented in the local wound tissues play a pivotal role in the orchestration of a dynamic pro-angiogenic process in which both local and remote events are involved.

**Figure 7:**
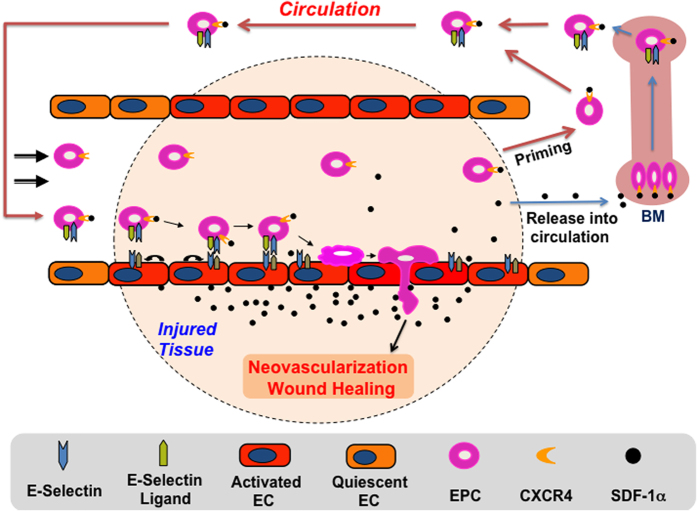
Illustration of a “double-lock” mechanism mediating EPC homing to ischemic wounds tissues for therapeutic neovascularization and wound repair. SDF-1α acts both locally and remotely to induce ischemic tissue endothelium and BMD-EPC to express E-selectin/ligands. Dual E-selectin/ligand pairs reciprocally expressed on activated endothelium and BMD-EPC mediate enhanced EPC-EC interactions and selective EPC homing.

Although SDF-1α causes both EC and EPC to express dual E-selectin/ligand pairs, the quantity of E-selectin and ligands, especially the activity of ligands (extent of glycosylation post translation) in EC and EPC, do not appear to be equal. SDF-1α-induced E-selectin ligands expressed on EPC showed less modification than those expressed on EC (a weaker binding activity of EPC than EC to HECA452 as showed in Fig. 2C,D). This may be attributed to the differences in microenvironment between wound endothelium (for EC modification) and BM niche or circulation (for EPC modification). The quantities and activity of enzymes responsible for glycosylation vary in different microenvironments and may not depend upon SDF-1α. In other words, expression and modification of E-selectin ligands may be determined by different mechanisms. Less activated E-selectin ligands on EPC could be important because this may not result in aggregation of EPC in circulation, especially under disruption of blood flow. However, such lowly activated E-selectin ligands on EPC may enable association with highly expressed E-selectin on activated endothelium in the swampy, low-flow capillaries in the ischemic tissues, where highly activated E-selectin ligands on EC can bind with the E-selectin on EPC to enhance EPC-EC interactions, which is essential to EPC homing, using what is here described as a “double-lock” mechanism (Fig. 7). In addition, the number of circulating EPC in blood is very low (approximately 1 EPC/μl in peripheral blood according to our previous studies^19^. It must be pointed out that the number of EPC depends upon the methods used to define EPC). Thus, the chance for EPC to meet/interact in the circulating blood is low. Therefore, all these factors, including less activated E-selectin ligands on EPC, low amount of EPC in circulating blood, and disruption of blood flow, prevent EPC from forming aggregates in the circulating blood. However, when circulating EPC arrive at the “swampy”, low-flow capillaries in the ischemic tissues, the local microenvironment (the quantities and activity of enzymes responsible for glycosylation of E-selectin ligands) may increase the activity of E-selectin ligands, which grants “double-lock” mechanism to work.

Roles of SDF-1α in retention of hematopoietic stem cells (HSC) in BM and recruited circulating mesenchymal stromal cells (MSC) in ischemic tissues have been reported^6^,^22^,^23^. SDF-1α-induced cell retention is ascribed to SDF-1α and its receptor CXCR4. Although it requires future study, our findings may suggest a potential role of up-regulated E-selectin/ligand pair on EPC in SDF-1α-induced retention of EPC in ischemic wound tissue. Because many tissue cells in ischemic limb and skin, including fibroblasts and inflammatory cells, express CD44 and/or CD162, E-selectin expressed on EPC can mediate cell-cell interaction between EPC and various tissue cells after EPC extravasate from blood vessels. Retention of EPC in ischemic tissue may be critical for neovascularization and tissue repair.

In summary, we report that SDF-1α elevated or therapeutically administered in ischemic wounded tissue can stimulate both local EC and BMD circulating EPC to express reciprocally E-selectin/ligand pairs and thereby enhance EPC-EC interactions. Our findings not only expand repertoire of SDF-1α and scope of SDF-1α-induced vascular adhesion molecules, but also identify a novel “double-lock” mechanism mediated by interaction of E-selectin/ligand pairs reciprocally expressed on the surface of both activated EC and EPC. Such a “double-lock” enhances and secures EPC-EC interactions by which to facilitate targeted EPC homing to ischemic wound tissue and subsequent EPC-associated neovascularization and repair responses. Further studies are warranted to address several important yet unanswered questions, for example, the molecular mechanisms underlying the regulation of E-selectin/ligand expression in EC and EPC by SDF-1α; the roles of each of these E-selectin ligands in mediating EPC-EC interaction.

## Methods

### Cells and mice

HMVEC, kindly provided by Dr. D. Fraker, University of Pennsylvania, Philadelphia, PA, were isolated from normal human dermis. Human EPC were purchased from NDRI (Philadelphia, PA). The same batch, but different passages of HMVEC and EPC were used in all experiments. Permission to use HMVEC and EPC was given from the ethics committee of University of Miami School of Medicine (IRB# 20080425) and complied to the Declaration of Helsinki. They were cultured as described previously^10^. *C57 BL6* mice were purchased from Charles River (Wilmington, MA), and *E-selectin*^*−/−*^ (*E-sel*^*−/−*^) (B6.129S4-sele^tmiDmil/J^), *Rosa26*^+/−^ (B6.129S7-GT (ROSA)26sor/J) which carry the *LacZ* gene and *NOD* (NOD/shilTJ) mice were from the Jackson Laboratory (Bar Harbor, ME). *Rosa26*^+/−^*;E-sel*^−/−^ mice were generated by mating *Rosa26*^+/−^ with *E-sel*^*−/−*^ mice and subsequently mating *Rosa26*^+/−^;*E-sel*^*+/−*^ with *E-sel*^*−/−*^ mice using standard breeding methods and validated by genotyping. For BMT experiments, 5 × 10^6^ bone marrow cells (BMC) from *Rosa26*^+/−^ or *Rosa26*^+/−^*;E-sel*^−/−^ mice were suspended in 200 μl of PBS and transplanted into γ-irradiated (850 rad) recipient *E-sel*^*−/−*^ or *C57 BL6* mice through tail vein 6 weeks before the experiment. Reconstitution of BM was validated 4–5 weeks after BMT by blood smear *X-gal* staining of recipient mice (>50% mononuclear cells in blood were LacZ^+^). Mice were maintained at the DVR animal facility under standard conditions and anesthetized for all surgical procedures by ketamine/xylazine (80/20 mg/kg, i.p.), and sacrificed in a CO_2_ chamber. All animal experimental protocols were approved by the Institutional animal care and use committee (IACUC) at the University of Miami. The methods were carried out in accordance with the approved guidelines.

### PCR array

*Extracellular Matrix & Adhesion Molecule PCR Array* quantitatively profiles 84 genes of adhesion molecules and ECM (PAHS-013Z, Qiagen, Valencia, CA). Subconfluent HMVEC and human EPC were stimulated with recombinant human (γh) SDF-1α protein (350-NS/CF, R&D Systems, Minneapolis, MN) vs. BSA at 100 ng/ml for 4 h (The dosage of γhSDF-1α (100 ng/ml) was pre-determined in our previous study^10^ based on the lowest concentration that could induce E-selectin expression in HMVEC). Total RNA was extracted using Trizol^®^ (Invitrogen, Grand Island, NY). cDNA was synthesized using RT^2^ First Strand Kit (Qiagen). PCR array was carried out according to the manufacturer’s protocol. The threshold cycle (Ct) values were used to plot a standard curve. All samples were normalized to the relative levels of *β-actin*, and results are expressed as fluorescence intensity in relative levels.

### Flow cytometry

Murine BMC was harvested as previously described^10^. Murine blood samples were immediately placed on ice at 4 °C. After red cell lysis, BMC were incubated with FITC-CD34, APC-KDR, and PE-E-selectin (#560238, 561252, #553751, BD Biosciences, San Jose, CA) or isotype-matched control Abs at 4 °C for 30 min using predetermined optimal concentrations of the test Abs. Cells were washed and analyzed using a FACScan flow cytometer (Becton Dickinson, San Jose, CA). EPC (CD34^+^/KDR^+^) were gated, counted, and analyzed for levels of E-selectin. Five thousand cells were analyzed per sample.

### ELISA

The concentrations of SDF-1α in the sera of mice were measured using Quantikine® SDF-1α ELISA kit (DY460, R&D Systems) based on the manufacturer’s protocol.

### Cell adhesion assay

HMVEC were cultured in 24-well plates to near confluency and stimulated with γhSDF-1α or BSA (100 ng/ml), and subsequently treated with soluble E-selectin (sE-sel, 10335-H08H, Sino Biological Inc., China) or BSA (2 μg/ml). 1 × 10^5^ Dil-Ac-LDL-labeled EPC, which were pre-cultured on a 2% agarose-coated plate to prevent adhesion to the plate^10^, stimulated with γhSDF-1α or BSA and subsequently treated with either E-selectin-neutralizing Ab (BBA1; R&D Systems) or isotype-matched control Ab. These were added to wells containing HMVEC monolayer for 30 min at 37 °C. Unbound EPC were washed out with PBS (twice) and adherent Dil-Ac-LDL-labeled EPC were measured by fluorescence scanner (GE Typhoon Trio, Piscataway, NJ).

### Immunoblotting and immunofluorescence

Immunoblotting was performed as described previously^10^,^19^. Subconfluent HMVEC and human EPC were stimulated with γhSDF-1α or BSA at 100 ng/ml for various periods as indicated. Cells were then harvested and subjected to immunoblotting as described^10^,^19^. Membranes were probed with Abs from Abcam (Cambridge, MA) to E-selectin (ab18981), CD162 (ab68143), CD44 (ab51037), ESL-1 (ab103439), and *β*-actin (AC-15). To assess CD44 expression in capillaries of ischemic wound tissues, sections were co-stained with FITC-CD44 and Alexa Fluor^®^594-CD31 (#103022, #102520, BioLegend, San Diego, CA) using immunofluorescence method as described previously^10^,^19^. In all immunofluorescence staining experiments, isotype-matched non-specific Ab was used as control (data not shown).

### Active form of E-selectin ligand binding assay

Subconfluent HMVEC and human EPC were stimulated with γhSDF-1α and BSA (100 ng/ml) for 16 h in 96-well plates. After washing with PBS, 200 ng/well of FITC-HECA452 (#321306, BioLegend) vs. FITC-BSA (P8779, Sigma-Aldrich, St. Louis, MO) were added into well and incubated at 37 °C for 30 min. After being washed twice with PBS, the plates were scanned using a fluorescence scanner and fluorescent imaging was recorded using a fluorescence microscope. Samples were triplicated and experiments were repeated three times.

### Laser Doppler perfusion imaging (LDI)

Limb perfusion was assessed daily using LDI (Periscan PIM II, Perimed AB, Sweden) as described previously^10^, Relative perfusion data are expressed as the ratio of the ischemic (right) to normal (left) limb blood flow.

### Induction of mouse hindlimb ischemic wounds

Creation of mouse hindlimb ischemia (right limb), cutaneous wounds and wound bed injection of γmSDF-1α protein (460-SD/CF, R&D Systems) was conducted as described previously^10^.

### Tissue-level detection of recruited BMD EPC

Frozen ischemic wound tissue sections were stained by *X-gal* (Fermentas, Canada) and HRP-anti-CD31 (ab28364, Abcam) and counterstained with nuclear fast red (Vector Labs) as described^10^. The number of EPC was quantified by counting LacZ^+^ cells in CD31^+^ vessels from 5 random high power fields (HPF, 20X) per section in at least 3 serial sections per wound sample (n = 6/group).

### Blood vessel perfusion and laser scanning confocal microscopy

Mouse blood vessels were labeled by live perfusion with DiI (D-282, Invitrogen/Molecular Probes) solution, and the vascular density was visualized by scanning the entire wound tissue to a depth of 200 μm, using laser scanning confocal microscopy (Vibratome (VT1000S, Leica Microsystems, Buffalo Grove, IL) as described^10^,^15^,^24^. Vessel density was quantified by assessing total number of Dil^+^ vessels normalized to the entire scanned wound area, using ImageJ software (Imaging Processing and Analysis in Java, National Institutes of Health, MD).

### Assessment of wound closure rate

Wound area was measured with daily digital photographs and images were analyzed using ImageJ as described^10^,^25^. The wound’s relative recovery rate is expressed as [(original wound area minus daily wound area)/(original wound area)] X 100.

### Statistical analyses

Statistical analysis of differences was performed using a 2-tailed Student’s *t*-test for two-group comparisons and two-way ANOVA test for multiple comparisons. Data are expressed as mean ± standard error of the mean (SEM). Values were considered statistically significant at *P* < 0.05. Statistical analyses were carried out with the SPSS 22.0 software package (SPSS, Inc., Chicago, IL, USA).

## Additional Information

**How to cite this article**: Liu, Z.-J. *et al*. SDF-1α-induced dual pairs of E-selectin/ligand mediate endothelial progenitor cell homing to critical ischemia. *Sci. Rep.* **6**, 34416; doi: 10.1038/srep34416 (2016).

## Supplementary Material

Supplementary Information
