# Supplementary material for: SDF-1α-induced dual pairs of E-selectin/ligand mediate endothelial progenitor cell homing to critical ischemia
Source: Sci Rep. 2016 Oct 7;6:34416. doi: 10.1038/srep34416 (PMC5054375; doi:10.1038/srep34416)
Supplement: Supplementary Information [file srep34416-s1.pdf]

SDF-1α-induced dual pairs of E-selectin/ligand mediate endothelial progenitor cell homing to critical ischemia

Zhao-Jun Liu[1], Runxia Tian[1], Yan Li[1], Leiming Zhang[1,2], Hongwei Shao[1], Cuixia Yang[1,3], and Omaida C. Velazquez[1,*]

[1] Department of Surgery, University of Miami School of Medicine, Miami, 33136, USA

[2] Yantai University, School of Pharmacy, Shandong 264005, China

[3] Department of Molecular Biology Laboratory, Shanghai Sixth People's Hospital, Shanghai Jiaotong University, Shanghai, 200233, China

[*] Corresponding author

Supplemental Table I

Fold changes of gene expression upon SDF-1α stimulation in human EPC

Gene name		*Fold change*
Collagen, type XII, alpha 1	+	2.64
Collagen, type VII, alpha 1	+	3.12
Versican	+	3.61
Catenin	+	5.10
Integrin, alpha 2	+	2.27
Integrin, alpha 3	+	2.22
Integrin, alpha 4	+	3.71
Integrin, alpha V	+	3.71
Integrin, beta 1	+	5.66
Laminin, alpha 3	+	2.66
Laminin, beta 3	+	97.68
Laminin, gamma 1	+	2.04
Matrix metallopeptidase 1	+	3.63
Matrix metallopeptidase 10	+	3.14
Matrix metallopeptidase 12	+	8.82
Selectin E	+	3.48
Sarcoglycan, epsilon	+	2.36
Secreted phosphoprotein 1	+	5.17
Transforming growth factor, beta-induced, 68kDa	+	2.06
Thrombospondin 1	+	4.44
Thrombospondin 2	+	3.76
Tenascin C	+	3.34
ADAM metallopeptidase with thrombospondin type 1 motif, 1	-	2.51
Integrin, alpha 8	-	2.07
Matrix metallopeptidase 9	-	2.39

Supplemental Figure 1

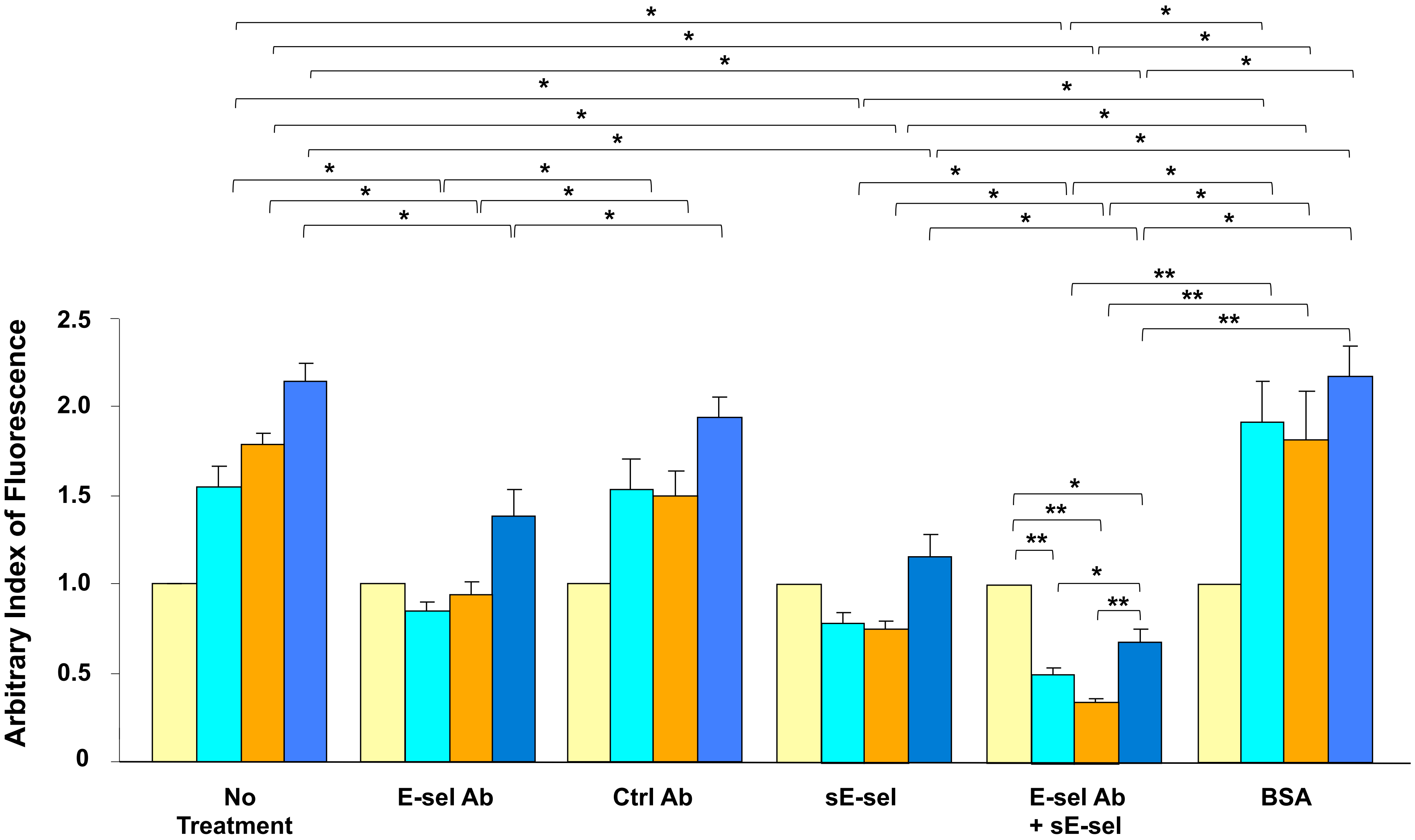

SDF-1α-induced E-selectin/ligands are responsible for mediating EC-EPC adhesion *in vitro*. SDF-1α increases adhesion of Dil-Ac-LDL+ human EPC to the HMVEC monolayer. Single or combined treatment of SDF-1α-stimulated HMVEC monolayers with sE-sel or treatment of SDF-1α-stimulated Dil-Ac-LDL+ EPC with E-selectin-neutralizing Ab can significantly inhibit EPC-HMVEC interaction. See Fig. 3 for the details of 4 combinations of EPC-HMVEC interactions and ANOVA test. * *P*<0.05; ** *P*<0.01.
